# Manipulating *osa-MIR156f* Expression by *D18* Promoter to Regulate Plant Architecture and Yield Traits both in Seasonal and Ratooning Rice

**DOI:** 10.1186/s12575-019-0110-4

**Published:** 2019-11-04

**Authors:** Qing Liu, Yi Su, Yunhua Zhu, Keqin Peng, Bin Hong, Ruozhong Wang, Mahmoud Gaballah, Langtao Xiao

**Affiliations:** 1 Hunan Provincial Key Laboratory of Phytohormones and Growth Development, Hunan Agricultural University, Changsha, 410128 China; 2 Southern Regional Collaborative Innovation Center for Grain and Oil Crops in China, Hunan Agricultural University, Changsha, 410128 China; 3 Hengyang Medical College, University of South China, Hengyang, 421001 China; 4 Rice Research and Training Center, Field Crops Research Institute, Agriculture Research Center, Giza, 33717 Egypt

**Keywords:** Seasonal rice, Ratooning rice, *Osa-MIR156f*, Plant architecture, Grain yield

## Abstract

**Background:**

Rice (*Oryza sativa* L.) feeds more than half of the world’s population. Ratooning rice is an economical alternative to the second seasonal rice, thus increasing the yield of ratooning rice is highly important.

**Results:**

Here we report an applicable transgenic line constructed through the manipulation of *osa-MIR156f* expression in rice shoot using the *OsGA3ox2* (*D18*) promoter. In seasonal rice, the D18–11 transgenic line showed moderate height and more effective tillers with normal panicle. In ratooning rice, axillary buds outgrew from the basal node of the D18–11 transgenic line before the harvest of seasonal rice. More effective tillers produced by the outgrowth of axillary buds contributed to the plant architecture improvement and yield increase. Additionally, it was found that osa-miR156f down-regulated the expression of tillering regulators, such as *TEOSINTE BRANCHED1* (*TB1*) and *LAX PANICLE 1* (*LAX1*). The expression of *DWARF10*, *DWARF27* and *DWARF53*, three genes being involved in the biosynthesis and signaling of strigolactone (SL), decreased in the stem of the D18–11 transgenic line.

**Conclusion:**

Our results indicated that the manipulation of *osa-MIR156f* expression may have application significance in rice genetic breeding. This study developed a novel strategy to regulate plant architecture and grain yield potential both in the seasonal and ratooning rice.

## Background

Rice (*Oryza sativa* L.) is the staple food of more than half of the world’s population which is expected to reach 9 billion by the year of 2050. To meet the needs of the projected population, at least a 40% improvement of crop yield will be needed by 2025 [1, 2]. Therefore, increasing crop yield is one of the most important goals in modern agriculture. The ideal plant architecture for rice, i.e. the phenotype of moderate height, enough effective tillers, large panicle and robust stems/roots, is crucial for high yield [3, 4]. In the past two decades, various genes regulating rice plant architecture related traits have been identified. For example, *Grain number 1a* (*Gn1a*) [5] and *ABERRANT PANICLE ORGANIZATION 1* (*APO1*) [6] regulate grain number. *Grain Size 3* (*GS3*), *Grain Weight 2* (*GW2*) and *SQUAMOSA-PROMOTER BINDING LIKE 13* (*SPL13*) regulate grain size [7–10]. *DENSE AND ERECT PANICLE 1* (*DEP1*), *SMALL PANICLE* (*SPA*) and *LAX PANICLE 1* (*LAX1*) control panicle size [11, 12]. *SPL14* promotes panicle branching and grain yield [13, 14]. *TEOSINTE BRANCHED1* (*TB1*) regulates lateral branching and represses the outgrowth of axillary buds [15]. *MONOCULM 1* (*MOC1*) controls rice branching and axillary meristem initiation [16]. *ELONGATED UPPERMOST INTERNODE1* (*EUI1*), *SEMI DWARF1* (*SD1*), *SLENDER RICE1* (*SLR1*), *GA-INSENSITIVE DWARF1* (*GID1*) and *GID2*, several genes being related to gibberellin (GA) biosynthesis/signal-transduction, jointly regulate plant height [17]. The successful identification of these genes involved in rice plant architecture regulation greatly promoted the elucidation of the underlying molecular mechanisms.

Strigolactones (SLs) regulate rice tiller development through interactions with tillering related genes. DWARF 53 (D53), the repressor of the SL signaling pathway, is able to directly interact with the N-terminal domains of miR156-controlled SPLs and represses the expression of *TB1* [13, 18, 19]. SPL14 interacts with MADS57, which directly binds to the CArG motif of the *DWARF14* (*D14*) promoter and suppresses *D14* transcription to control the outgrowth of axillary buds in rice [20]. *LAX1* and *Rice Leafy Homolog1* (*RFL*)*,* being highly co-expressed with *SPL7*, *SPL14*, and *SPL17*, are down-regulated in the panicles of the *osa-MIR156* overexpression line [11, 21, 22]. SPL14 is able to bind to the *LAX1* promoter, which implies that *LAX1* may also be directly regulated by *SPL14* [23]. The expression of *Rice TFL1/CEN homolog1* (*RCN1*) is suppressed by MADS34 [24, 25]. *MADS34*, *SPL*, *LAX1* and *RFL* are down-regulated in the *osa-MIR156b* and *osa-MIR156h* overexpression line [26].

Several studies focused on the important regulators for rice plant architecture. Among them, microRNAs show immense application potential in genetic breeding because of their flexible and precise regulation of tillering and panicle branching. miR156 targets SPLs and regulates plant growth and development [27]. Furthermore, miR156 and *SPLs* are involved in plant embryogenesis [28], shoot maturation [29], flowering control [30], phase change [27, 31–34], biomass production [31, 35], panicle cell death [36], and crown root development [37]. In rice, ten *osa-MIR156* genes produce five mature osa-miR156 sequences and the overexpression of *osa-MIR156* produces more tillers [26]. SPL14 defines the plant architecture by decreasing tiller number and increasing plant height/panicle branch number, thus shows great potential for genetic breeding [13, 14]. Interestingly, SPL14 mRNA contains a recognition site for miR156 and its spatiotemporal expression is strictly controlled by miR156. Our previous study also found that a high level of *osa-MIR156f* in rice caused a dwarf and multi-tillering phenotype [38]. Additional regulatory networks, such as the miR529/SPL, miR172/AP2 and miR156/miR159 pathways, also influence rice tillering and panicle branching [23, 34, 39]. Moreover, SLs suppress shoot branching by inhibiting the outgrowth of axillary buds through the D53 repressor signaling pathway. D53 interacts with IDEAL PLANT ARCHITECTURE1 (IPA1) in vivo and in vitro to suppress the transcriptional activation activity of SPL14. SPL14 functions as a direct downstream component of D53 in regulating tiller number and SL-induced gene expression. SPL14 may directly bind to the *D53* promoter and plays a critical role in the feedback regulation of SL-induced *D53* expression [40]. Although the miR156/SPL14 pathway is important in rice tillering, constitutive overexpression lines of *osa-MIR156* produce a severely defective phenotype, with the tillers and panicles being totally ineffective.

Ratooning rice is an economical alternative to the second seasonal rice in subtropical and temperate zones, where the light and temperature are not enough for double cropping cultivation [41]. Compared to the second seasonal rice cultivation, ratooning rice may significantly decrease the production costs by reducing the input of labor force, fertilizer, pesticides and herbicides [42]. Furthermore, ratooning rice results in better grain quality and less field pollution [42, 43]. Thus the research of ratooning rice is of great significance.

Ratooning rice develops from the regenerated tillers on the stubble left behind the first seasonal rice harvest. Meanwhile, the different origin points of ratoon tillers on the main-crop stubble show different yield potential since ratoon panicles decrease in size as the point of origin changes acropetally (from the base upward) [41]. Similar to the first seasonal rice, the early ratoon tillers originated from the internodes are the main contributors for ratooning rice yield. In fact, compared to seasonal rice, grain yield in ratooning rice is more dependent on the fertile tiller number per plant and fertile grain number per panicle [44].

Guiding precise gene expression in plant tissues using tissue specific promoters is feasible to reduce the negative effects of overexpression. Recently, it had reported a modification in the expression of *IPA1* increases disease resistance against bacterial blight and substantially increases the yield potential in rice by that using the bacterium-inducible promoter of *OsHEN1* [45]. In this study, *osa-MIR156f* was specifically expressed in the rice stem under the control of the promoter of a GA biosynthesis gene, *GA3ox2* (*D18*) [46, 47]. Compared to the wild type, the transgenic line presented moderate height, more effective tillers, and increased grain yield both in seasonal and ratooning rice. The manipulation of *osa-MIR156f* expression may have application significance in rice genetic breeding. Moreover, it was hypothesized that miR156f may control tillering through manipulating the spatiotemporal expression of *OsTB1*, *OsLAX1* and *OsD53*.

## Results

### Expression Pattern of *osa-MIR156f* in Rice

**Fig. 1:**
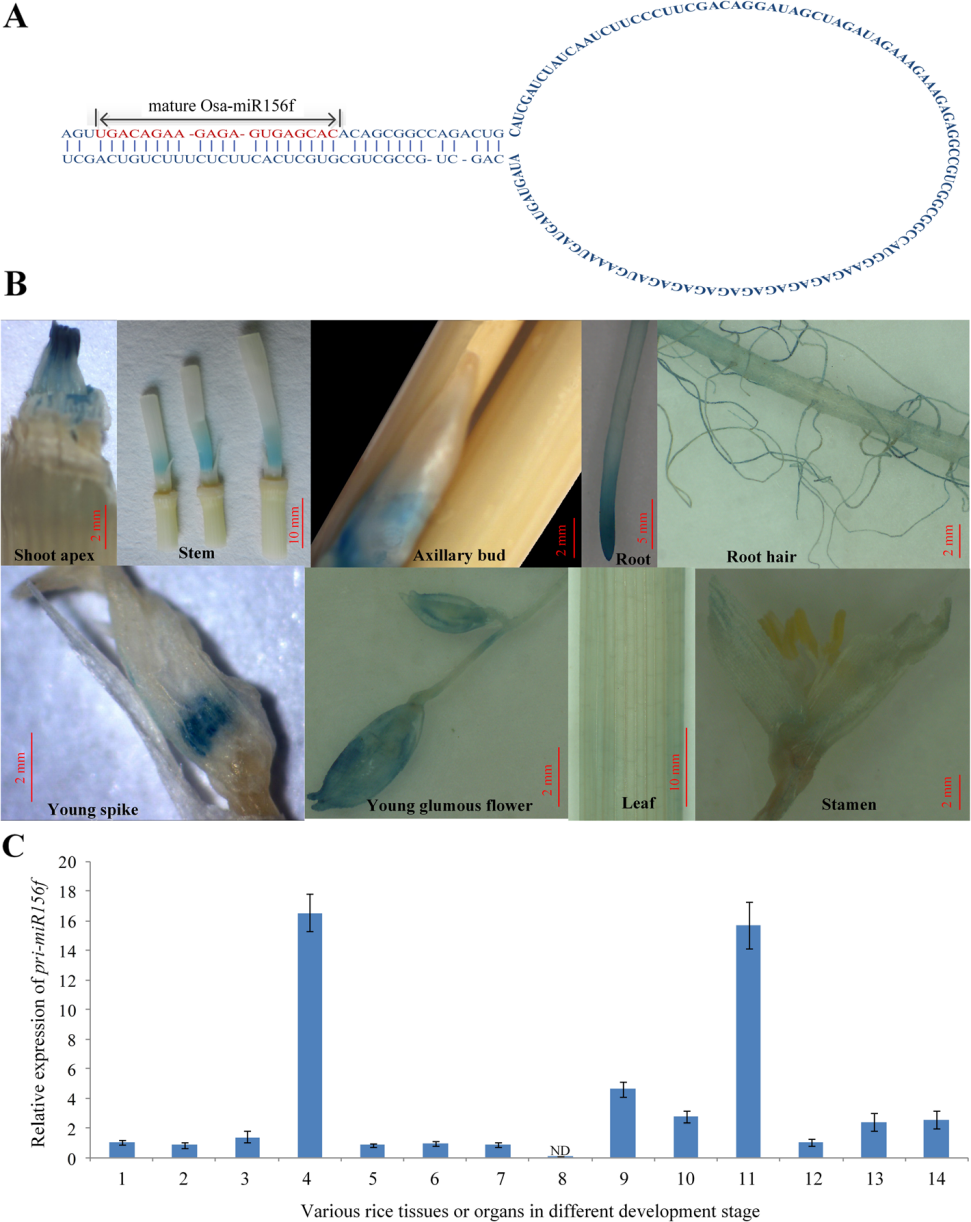
Expression pattern of *osa-MIR156f*. **a** The typical hairpin structure of pre-miR156f and the 20 nt of the mature miR156f. **b** Localization of GUS activity driven by the *osa-MIR156f* native promoter in different tissues. The sampling dates for tissues were as follows: shoot apex at the 3-week-old seedling stage, stem at the flowering stage, axillary bud at the heading stage, root at the 3-week-old seedling stage, root hair at the tillering stage, young spike at the heading stage, young glumous flower at the panicle booting stage, leaf segment at the heading stage and stamen at the flowering stage. **c** Relative expression of *pri-miR156f* in various rice tissues or organs: 1, leaf lamina at tillering stage; 2, leaf sheath at the tillering stage; 3, shoot apex at the tillering stage; 4, stem segment (about 0.5 cm) above the node at the flowering stage; 5, axillary bud at the tillering stage; 6, young spike at the heading stage; 7, young glumous flower at the heading stage; 8, stamen (ND: not detected); 9, basal node at the heading stage; 10, root tip (about 0.5 cm) at the heading stage; 11, 3-week-old leaf; 12, 3-week-old shoot; 13, 3-week-old basal node; 14, 3-week-old root. The ΔΔCt method was used to calculate the relative gene expression. Error bars indicated the standard deviation (SD). Three independent biological replicates were analyzed (*n* = 3)

Two copies of *osa-MIR156f* are adjacently located in two large repeat fragments on chromosome 8 of Nipponbare. *Pre-miR156f* can form a typical hairpin, and the mature osa-miR156f is a 20 nt fragment (Fig. 1a). In previous study, we have identified that *osa-MIR156f* plays important roles in rice tillering and plant architecture [38]. To further understand the detailed function of *osa-MIR156f*, we obtained 21 independent transgenic lines by harboring *osa-MIR156f pro::GUS* (β-glucuronidase) fusions from Nipponbare and observed the expression pattern of *osa-MIR156f* during rice growth and development. Histochemical analysis showed that the *GUS* expression was spatiotemporally restricted. High *GUS* expression was found in the young tissues, such as the shoot apex, axillary bud, young spike and young glumous flower (Fig. 1b). Strong GUS activity was associated with elongating zones in the stem (Fig. 1b). The GUS staining was also detected in root hair, root tip, young spike and glumous flower (Fig. 1b). In the axillary tiller buds, the GUS staining only appeared in bud on outgrowable unelongated basal internodes, but not in dormant buds on elongated internode (Fig. 1b, Additional file 1: Supplemental file S4). In addition, *GUS* expression was detected in leaf lamina at tillering stage and flag leaf at heading stage (Additional file 1: Supplemental file S4 D and E). To further analyze the expression pattern of *osa-MIR156f*, the level of *pri-miR156f* was relatively quantified by RT-qPCR in young tissues of the wild type at different developmental stages. The expression of *osa-MIR156f* was detected in young tissues except stamen (Fig. 1c). Young stem segment (about 0.5 cm) above the node at the tillering stage, basal node at heading stage, and 3-week-old leaf showed higher level of *pri-miR156f* than other young tissues (Fig. 1c).

### Manipulation of *osa-MIR156f* Expression in Rice Shoot by the *D18* Promoter

**Fig. 2:**
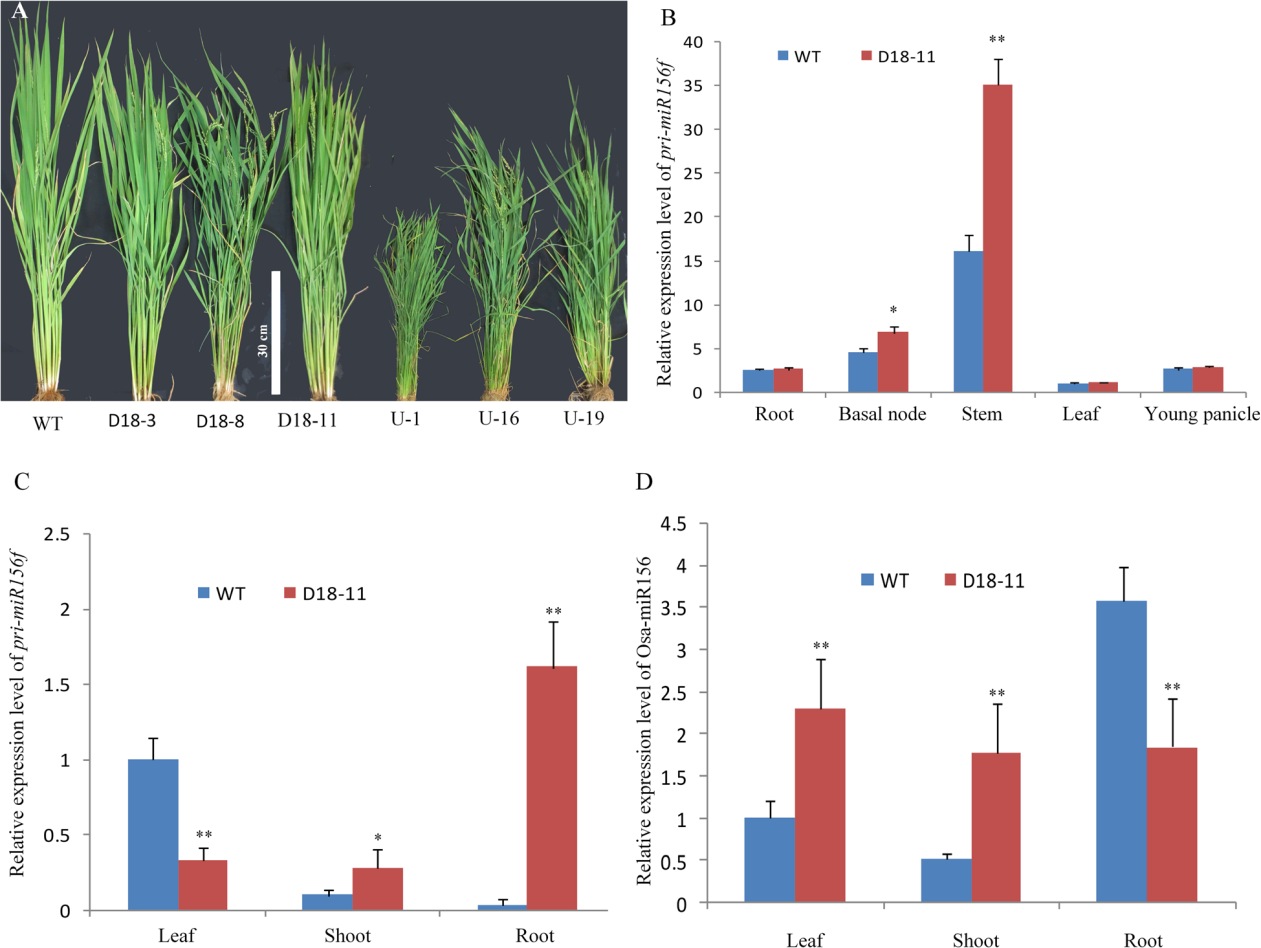
Expression of *osa-MIR156f* in transgenic rice. **a** Phenotype of transgenic rice lines including three *D18p::osa-MIR156f* lines (D18–3, D18–8 and D18–11) and three *UBQpro*::*osa-MIR156f* lines (U-1, U-16 and U-19). **b** Expression analysis of *pri-miR156f* in different tissues of D18–11 and the wild type (WT) rice at the heading stage. **c** Expression analysis of *pri-miR156f* in different tissues of D18–11 and the WT rice at 3-week-old seedling stage. **d** Mature osa-miR156 level in different tissues of D18–11 and WT rice at 3-week-old seedling stage. The ΔΔCt method was used to calculate the relative gene expression. Error bars indicated the standard deviation (SD). Three independent biological replicates were analyzed (*n* = 5). Asterisks indicate a significant difference to WT rice based on a *t* test. *: significant difference compared to WT rice at a 5% level (*P* < 0.05); **: significant difference compared to WT rice at a 1% level (*P* < 0.01)

The constitutive overexpression of *osa-MIR156* in rice may sharply downregulate the level of *SPL14,* and then bring about severe defects in tillering and panicle development [23, 26] (Fig. 2a). In the current study, we applied different promoters to drive the expression of *osa-MIR156f* in rice and observed the subsequent effects on rice plant architecture respectively. In our previous research, *osa-MIR156f* was constitutively overexpressed through the *Ubiquitin1* promoter in Nipponbare and more than 35 independent lines with similar architecture were screened [38]. Among them, U-16 showed severely defective phenotypes, such as dwarf architecture, distinct reduction in filled grain numbers and an obvious increase in ineffective tillers (Fig. 2a). These defects in the rice grain of the *UBQpro::osa-MIR156f* lines indicated that the high osa-miR156 may be deleterious to the reproductive development.

Previous researches indicated that expression of *D18* was observed around the shoot apex [48]. *OsGA2ox1* ectopic expression in shoots by the promoter of *D18* resulted in a semi-dwarf phenotype with normal flower and grain development [46]. In order to reduce the negative effects of overexpressed *osa-MIR156f* on rice growth and development, the *D18* promoter was employed to drive the expression of *osa-MIR156f* in rice. We screened 30 independent lines with similar phenotype in plant architecture from 77 candidate transgenic lines. Comparing to *UBQpro::osa-MIR156f* transformants and WT, most of the *D18pro::osa-MIR156f* lines did not show defective phenotypes (Fig. 2a, Additional file 1: Supplemental file S5). Therefore, we selected a typical *D18pro::osa-MIR156f* transgenic line (named D18–11) as the material in this study. D18–11 produced more effective tillers than the wild type. At the heading stage, gene expression analysis showed that the transcriptional levels of *pri-miR156f* in stems and basal nodes of D18–11 were higher than that of the wild type, but no differences were observed in the roots, leaves and panicles (Fig. 2b). The levels of osa-miR156 and *pri-miR156f* were also examined in 3-week-old seedlings. Results showed that the *pri-miR156f* contents and mature osa-miR156 levels in different organs of D18–11 were not always consistent (Fig. 2c, d). Although the *pri-miR156f* level in roots was increased compared to the wild type, osa-miR156 level was lower in D18–11 than that in the wild type. The level of *pri-miR156f* in leaves was down-regulated, but the osa-miR156 level was increased in D18–11 compared to the wild type. The level of *pri-miR156f* and osa-miR156 in the shoot was significantly up-regulated in D18–11 (Fig. 2c, d). These results indicated that the *D18* promoter may precisely manipulate the tissue-specific expression of *osa-MIR156f* in stem.

### *D18pro::osa-MIR156f* Rigorously Affected Tillering Related Gene Expression and SL Level

**Fig. 3:**
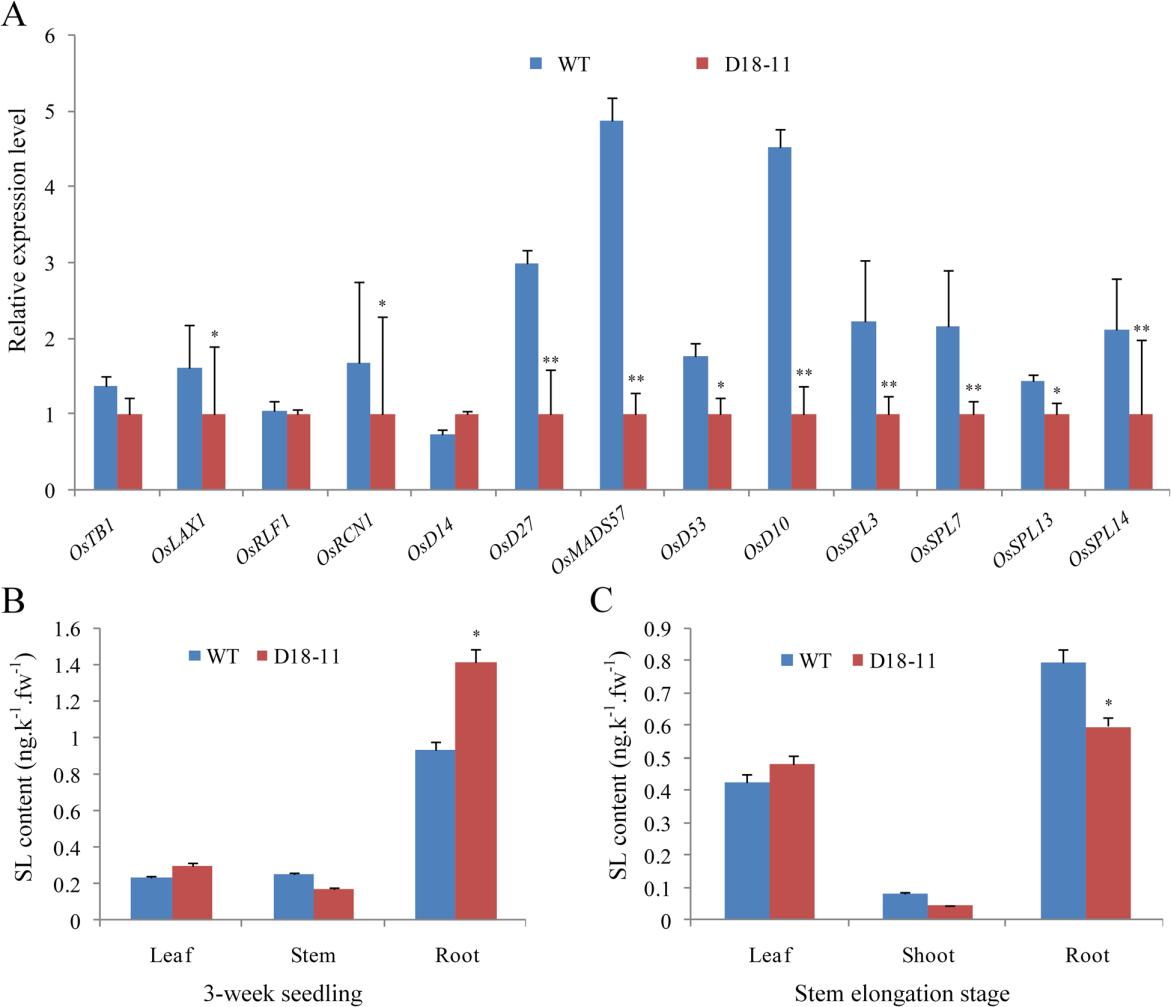
Effects of *osa-MIR156f* on gene expression and SL synthesis. **a** Expression of the tillering and branching related genes in the D18–11 transgenic rice and the wild type (WT) rice; **b** SL contents in 3-week seedling stage; **c** SL content at the stem elongation stage (about 70 days after germination). The data calculations are normalized to the internal rice *tubulin* gene. The ΔΔCt method was used to calculate the relative gene expression. Error bars indicated the standard deviation (SD). Three independent biological replicates were analyzed (*n* = 3). Asterisks indicate a significant difference to WT rice based on a *t* test. *: significant difference compared to WT rice at a 5% level (*P* < 0.05); **: significant difference compared to WT rice at a 1% level (*P* < 0.01)

**Fig. 4:**
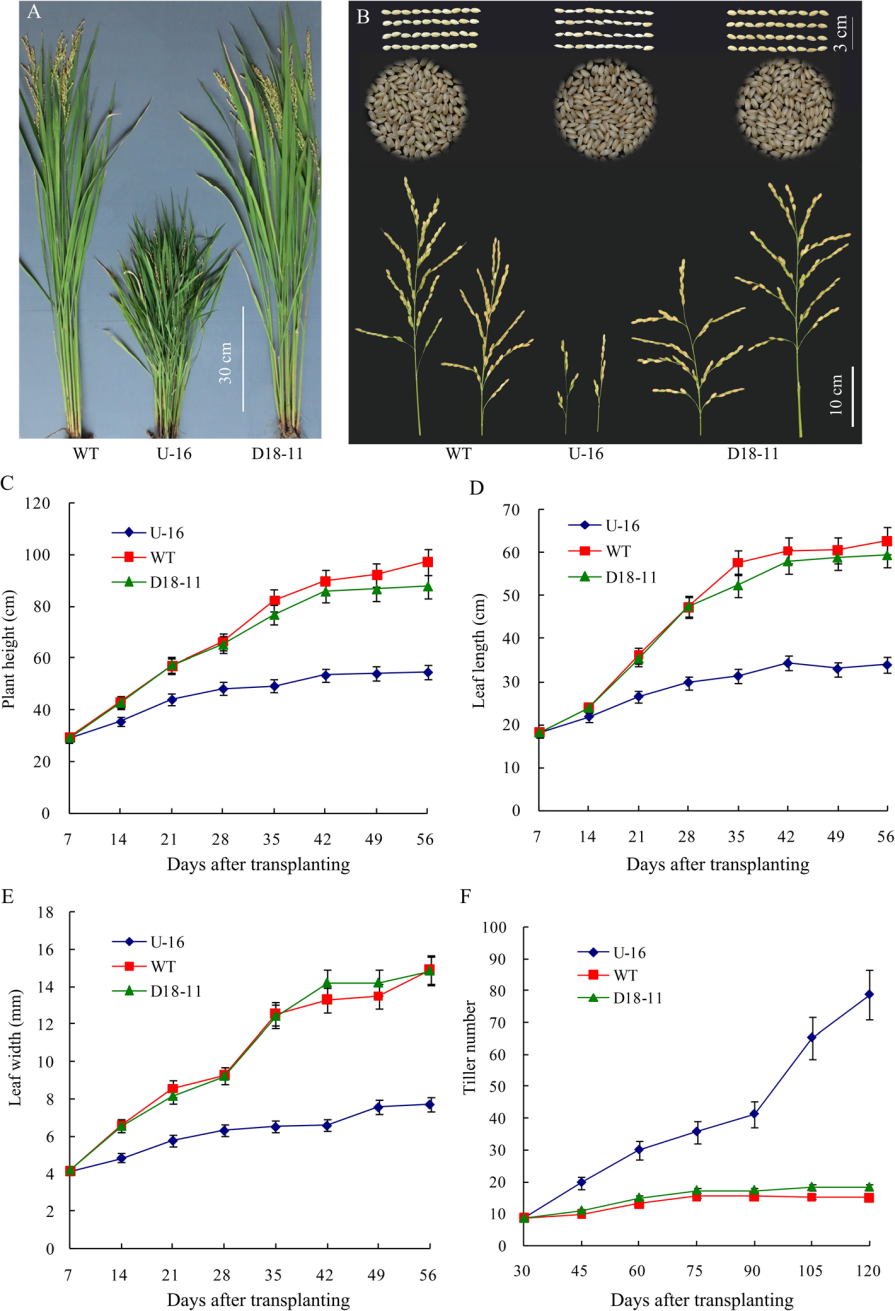
Effects of *osa-MIR156f* on plant architecture in the seasonal rice. **a** Phenotype of 110 days old seasonal rice the in the wild type, U-16, and D18–11; **b** Effects of *osa-MIR156f* on the panicle and grains in the wild type, U-16 and D18–11; For the wild type, U-16 and D18–11, the grain lengths (cm) were 0.721 ± 0.035, 0.712 ± 0.027 and 0.715 ± 0.058 respectively, and the grain width (cm) were 0.311 ± 0.046, 0.285 ± 0.046 and 0.315 ± 0.024 respectively; **c**-**f** Effects of *osa-MIR156f* on the plant height, leaf width, leaf length and tiller number in seasonal rice. Three independent biological replicates were analyzed (*n* = 50). Error bars indicated the standard deviation (SD)

*osa-MIR156f* was driven to express in the stem by the *D18* promoter to regulate rice tillering. To confirm the downstream regulators, some tillering and branching related genes were selected for further investigation by RT-qPCR at the tillering stage (Fig. 3). The target genes (including *OsSPL3/7/13/14*) of osa-miR156 were down-regulated in the stem of D18–11 (Fig. 3a, Additional file 1: Supplemental file S6). Previous research found that OsSPL13 positively regulates cell size in the grain hull, resulting in enhanced rice grain length [10]. However, no significant differences in grain size (including length, width and 1000-grain weight) were detected between D18–11 and the wild type although the expression of *OsSPL13* was slightly down-regulated (Table 1, Fig. 4b). In addition, *OsTB1* was slightly down-regulated, and both the transcriptional levels of *OsLAX1* and *Rice OsRCN1* were significantly reduced (*P* < 0.05).

**Table 1:** Yield traits of different rice lines in the seasonal crop

Yield traits	WT	D18–11	U-16
Tiller number per plant	7.4 ± 0.9	9.1 ± 2.4*	46.8 ± 15.6**
Main panicle length (cm)	22.49 ± 1.61	21.70 ± 0.76 NS	7.94 ± 1.04**
Total grains per main panicle	126.0 ± 17.1	127.0 ± 10.9 NS	10.7 ± 2.2**
Filled grains per main panicle	97.1 ± 12.4	85.6 ± 13.3*	7.5 ± 2.0**
Filled grain percentage (%)	82.3 ± 5.0	75.2 ± 14.8*	70.9 ± 13. 9**
1000-grain weight (g)	24.3 ± 0.7	22.7 ± 0.5 NS	18. 2 ± 0.1**
Grain yield per plant (g)	14.6 ± 1.3	18.1 ± 3.6**	2.9 ± 0.6**

Values are the means ± SD of three biological replicates (*n* = 150). Asterisks indicate a significant difference compared to the wild type (WT) rice based on a *t* test. **: significant difference compared to WT rice at 1% level (*P* < 0.01); *: significant difference compared to WT rice at 5% level (*P* < 0.05); NS: no significant difference

Interestingly, *osa-MIR156f* significantly affected the SL signaling and biosynthesis pathways. *OsD14*, *OsDWARF10* (*D10*), *OsDWARF27*(*D27*), *OsD53* and *OsMADS57* were down-regulated in the stem of D18–11 (Fig. 3a). SL contents were significantly different at the 3-week-old seedling stage and stem elongation stage in D18–11 and the wild type. In 3-week-old seedlings, lower SL level was detected in shoots and roots of D18–11 in comparison to the wild type (*P* < 0.05) (Fig. 3b). SL content differences in leaves were not significant between D18–11 and the wild type. At the stem elongation stage (about 70 days after germination), SL in D18–11 stems was lower than that in the wild type (*P* < 0.05), but SL in D18–11 roots was significantly increased (*P* < 0.01) (Fig. 3c). These results indicated that osa-miR156 may affect SL synthesis or SL homeostasis in rice.

### *D18pro::osa-MIR156f* Demonstrated Improved Architecture Characteristics and Increased Rice Yield

In this study, semi-dwarf transgenic rice plants (D18–11) with more effective tillers were obtained (Fig. 4a-b). To further analyze the influence of *osa-MIR156f* on rice development, the morphological indexes and grain yield were investigated. At all investigated time points, the height and leaf length of the transformant U-16 were both shorter than that of the wild type, and leaf width was narrower than the wild type (Fig. 4c-f). The average plant height of U-16 was less than 50 cm but the average tiller number was more than 40, accompanied by narrower and shorter leaves compared to the wild type (Fig. 4c). Most transformants of D18–11 plants were able to grow up to over 90 cm in height. No severe dwarf lines similar to the U-16 transformants were observed. The tiller number of D18–11 and the wild type did not increase from 60 to 120 days after transplanting (DAT), while U-16 went on to initiate and formed many non-productive tillers in the same period (Fig. 4c-f).

**Fig. 5:**
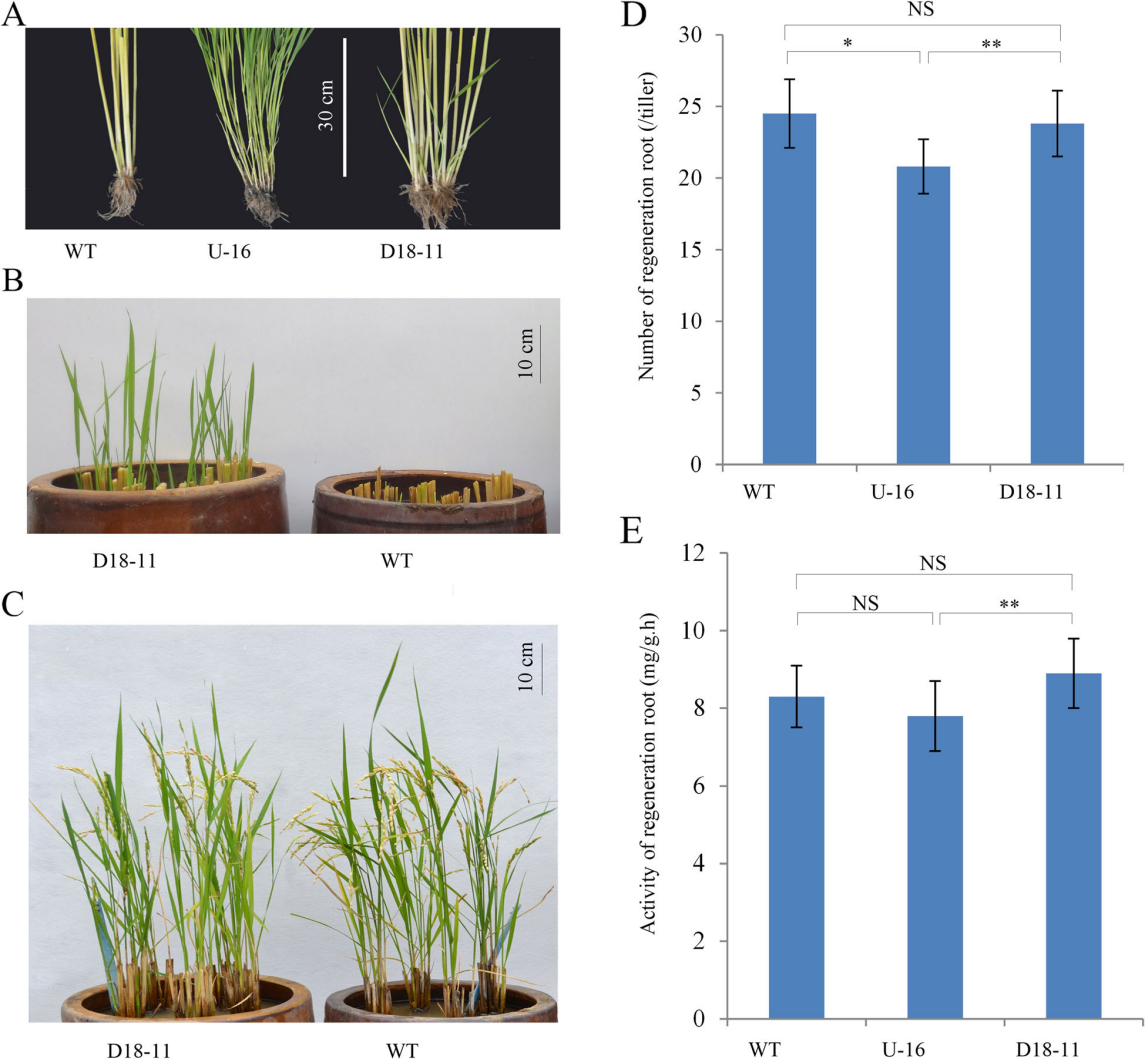
Effects of *osa-MIR156f* on the ratooning rice. **a** Tillering phenotypes in the seasonal rice. **b** Outgrown axillary buds of D18–11 at 5 days after seasonal rice harvest. **c** Phenotypes of the ratooning rice. **d** Number and **e** activity of regenerated roots in ratooning rice. Three independent biological replicates were analyzed (*n* = 50). Error bars indicated the standard deviation (SD). Asterisks indicate a significant difference based on a *t* test. *: significant difference compared to the WT rice at a 5% level (*P* < 0.05); **: significant difference compared to the WT rice at a 1% level (*P* < 0.01); NS: no significant difference

After the heading stage, the axillary buds both in U-16 and D18–11 displayed a remarkable difference from the wild type. All axillary tiller buds remained dormant and rarely outgrew in the wild type, whereas nearly all axillary buds outgrew and eventually formed tillers in U-16. Some axillary buds in D18–11 outgrew and formed young tillers around the unelongated basal nodes, but the axillary buds on the elongated nodes remained dormant as in the wild type (Fig. 5a).

As a complex trait in rice, grain yield is multiplicatively determined by three components: the number of panicles, number of grains per panicle and grain weight [49]. Compared to the wild type, more tillers with shorter panicles and fewer filled grains were produced in U-16 in the pot experiment (Table 1). The yield related traits were also noticeably impaired, thus the actual grain yield was significantly reduced in U-16 (Table 1). In contrast, D18–11 showed no deleterious effects on reproductive organs, and no significant difference was observed in main panicle length and filled grain percentage between the D18–11 and the wild type. Due to more effective tillers, D18–11 had an increased grain yield of up to 18.1 g per plant compared to 14.6 g per plant in the wild type, suggesting the possibility for improving plant architecture and yield potential. The grain yield per plant in D18–11 was further evaluated by field trial. D18–11 showed no difference to the wild type in main panicles length, but effective tiller number and grain yield per plant were distinctly increased (Table 1). These results indicated that the stem-specific expression of *osa-MIR156f* driven by the *D18* promoter may create an improved plant architecture with more effective tillers and no panicle defects, although the constitutive overexpression of *osa-MIR156f* resulted in seriously impaired reproductive development.

### *D18pro::osa-MIR156f* Transgenic Plants Showed Superior Ratooning Performance

In the cultivation practice of ratooning rice, the outgrown buds from the last two nodes are easy to form effective tillers, but the buds from the basal node are considered inferior because of their longer growth time. Fortunately, the axillary buds of D18–11 around the basal nodes would outgrow in advance before the harvest of seasonal rice (Fig. 5a). This ensured that early outgrown buds could develop into effective tillers and contribute to the grain yield of ratooning rice within 50 to 60 days (Fig. 5b-c). Therefore, the outgrown buds in advance were useful for tapping the potential of ratooning rice yield (Table 2). To evaluate whether those earlier sprouting axillary tiller buds contributed to the ratooning rice yield, we investigated ratooning performance related agronomic traits of D18–11 and the wild type after the harvest of seasonal rice. D18–11 showed moderate plant height, more effective tillers, and higher grain yield per plant compared to the wild type (Table 2). In addition, both the number and activity of newly regenerated roots per tiller showed no significant difference between D18–11 and the wild type in ratooning rice, but the root activity of U-16 was significantly decreased compared to D18–11 and the wild type (Fig. 5d and e, Table 2). The results indicated that manipulating the expression of *osa-MIR156f* in stem using the *D18* promoter did not affect both the quantity and activity of ratooning rice roots.

**Table 2:** Agronomic traits of D18–11 and wild type in seasonal and ratooning crop

Traits	WT		D18–11	
	Seasonal	Ratooning	Seasonal	Ratooning
Plant height (cm)	87.6 ± 4.4	53.37 ± 1.9	84.5 ± 6.2*	50.62 ± 4.8 NS
Tiller number per plant	6.6 ± 1.7	7 ± 1.4	9.1 ± 1.2**	8.7 ± 2.6*
Main panicle length (cm)	20.68 ± 1.84	11.38 ± 2.58	20.04 ± 1.73 NS	11.33 ± 2.40 NS
1000-grain weight (g)	24.38 ± 0. 21	24.22 ± 0. 19	22.80 ± 0.55 NS	23.89 ± 0. 31NS
Grain yield per plant (g)	12.26 ± 2. 49	2.54 ± 0.40	16.82 ± 2. 93**	2.68 ± 0.23*
Root activity (mg/g.h)	/	8.3 ± 0.8	/	8.9 ± 0.9

Values are the means ± SD of three biological replicates (*n* = 50). Asterisks indicate a significant difference compared to wild type (WT) rice based on a *t* test. *: significant difference compared to WT rice at 5% level (*P* < 0.05); **: significant difference compared to WT rice at 1% level (*P* < 0.01); NS: no significant difference. /: represented no dada collected

## Discussion

### *osa-MIR156f* Systematically Regulates Expression of Tillering Related Genes in Shoot

**Fig. 6:**
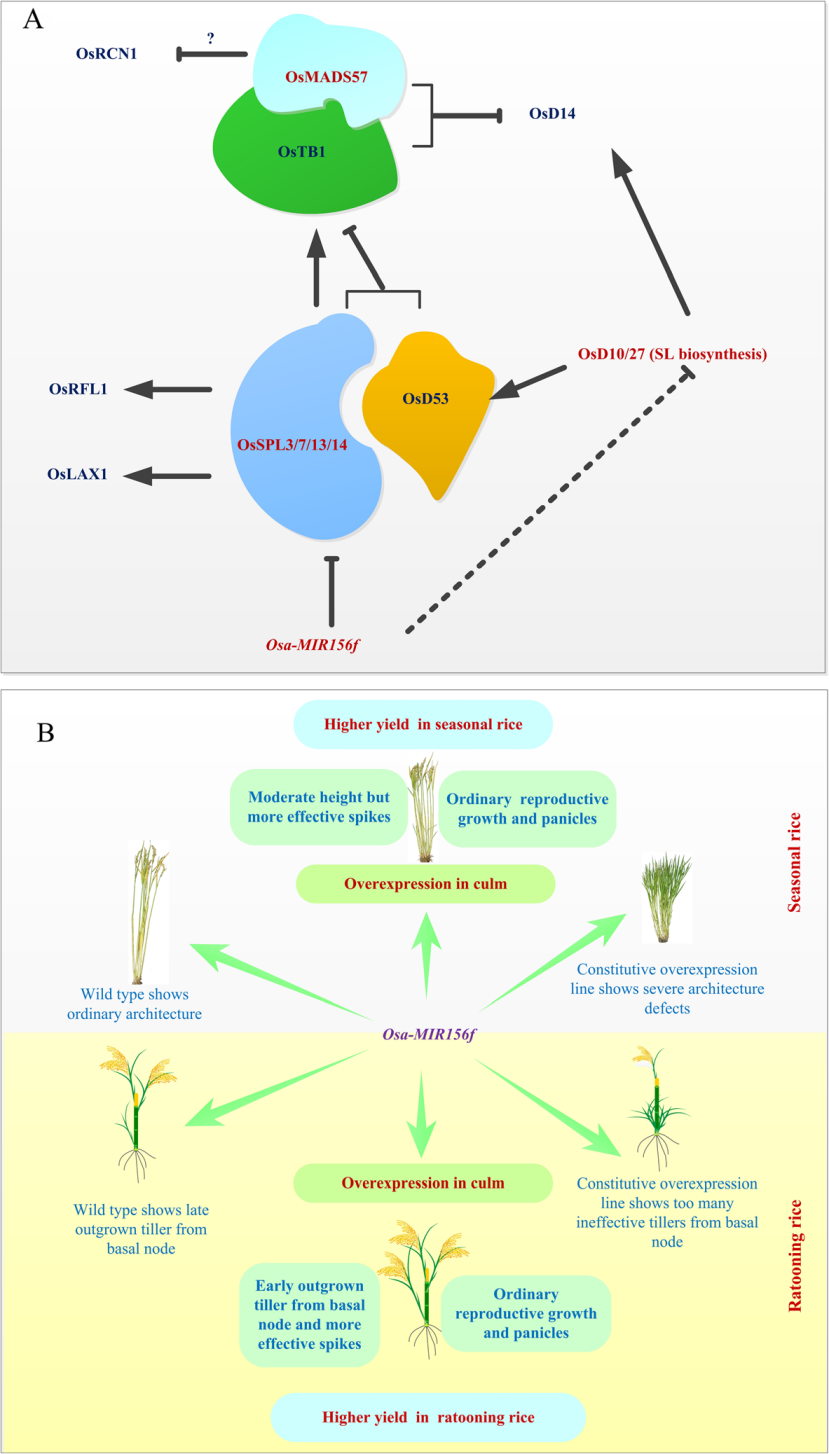
Effects of *osa-MIR156f* on (**a**) expression of tillering and branching regulators and (**b**) plant architecture in rice

Rice tillering and branching are largely controlled through the miR156/SPL pathway. Previous studies indicated that miR156 promotes tiller branching, but negatively regulates the activity of the inflorescence meristem [26, 38]. Recently, more regulators have been identified and more interactions among D53, SPL3/17, TB1, and LAX1/RFL have been elucidated in this pathway [11, 20–23, 40]. Many rice panicle development related genes, including *MADS34*, *SPLs*, *LAX1* and *RFL*, are down-regulated in *osa-MIR156* overexpression plant [23]. The expression regulation of tillering/branching related regulators was briefly summarized in Fig. 6a. osa-miR156 targets *SPLs* and reduces their transcriptional level. The transcription of *TB1* is activated by SPL3/17, but represses by D53 [19]. MADS57 and TB1 can be assembled to a heterodimer and then suppress the expression of *D14* [20]. *LAX1* and *RFL* are highly co-expressed with *SPLs* [22]. The expression of *RCN1* is suppressed by MADS34, but further evidence is still needed [24, 25].

In this study, a transgenic rice line was generated by harboring *osa-MIR156f pro::GUS* to observe the expression pattern of *osa-MIR156f*. High *GUS* expression was found in the young tissues. *Osa-MIR156f* was mainly expressed in the root tip, elongating zone and root hair (Fig. 1b). The expression of tillering related genes in the transgenic line was detected and their relationship was analyzed. The stem specific expression of *osa-MIR156f* driven by the *D18* promoter extremely down-regulated the transcriptional levels of *MADS57*, *D10*, *D27* and *SPLs* in the stem (Fig. 3a). A previous research found that *MADS57* expression was higher at the tillering and stem-elongation stages than at other stages in rice [20]. A high transcriptional level of *MADS57* was found in the sheaths and leaves, but weak signals were observed in the culm [20]. *osa-MIR156f* in stem may further decrease the level of *MADS57* mRNA and then promote the expression of *D14* (Fig. 3a). Slightly higher level of D14 may cause moderate height and more effective tillers, but not severe dwarfism and excessive ineffective tillers in D18–11 (Fig. 2a). *osa-MIR156f* in rice stem has little effect on the transcriptional levels of *TB1*, *LAX1* and *RFL1* because these three genes are mainly expressed in the panicles. Unexpectedly, it was found that *RCN1* was down-regulated in the stem, but another study indicated that *RCN1* was up-regulated in panicles of miR156 overexpression plants [23]. Furthermore, it was found that *MIR156f* significantly inhibited the expression of *D10* and *D27,* which encode two important enzymes in SL biosynthesis. Moreover, the SL contents were significantly decreased both in the shoot and stem of D18–11. osa-miR156 may regulate SL biosynthesis in addition to signaling through an unknown pathway (Fig. 6a). Overall, the plant architecture of D18–11 is the result of the systematical regulation by multiple regulators, including *SPLs*, *MADSs* and SL signaling genes.

### Manipulation of *osa-MIR156f* Shaped Plant Architecture and Yield Traits both in Seasonal and Ratooning Rice

For an individual plant, the rice grain yield is determined by the number of effective tillers (panicles), number of grains per panicle, and grain weight [49]. In addition, plant architecture is also a very important determinant of yield per unit area. To obtain high rice gain yield, breeding efforts in the past 50 years have brought about two great advancements in yield through the application of the semi-dwarf gene and development of hybrid rice. Super-rice breeding programs have achieved great success. Identification of *IPA* alleles has been a critical objective for enhancing global food security. Among them, IPA1 dampens tiller branching, but increases panicle branching and grain weight together with strengthening culms [13, 14]. *IPA* traits can be fine-tuned by manipulating *IPA1* expression, thus an optimal *IPA1* expression may lead to an ideal plant architecture and yield, which demonstrates a practical approach to the efficient design of elite super rice varieties [50]. It has been demonstrated that the miR156/SPL pathway manipulates a large range of processes underlying plant growth and development, including embryonic patterning, phase change and plant architecture establishment [51]. As the controller of SPLs, the activities of miR156 are so influential that the constitutive overexpression of *osa-MIR156* led to severe defects in tillering and branching [26, 38] (Figs. 1 and 6b). More tillers with shorter panicles and fewer grains were produced in the U-16 plants. The U-16 plants showed a drastic reduction in panicle branches and filled grain number per panicle. The yield traits (as mentioned above) were also noticeably impaired in the U-16 line (Table 1, Fig. 6b). Fortunately, the *osa-MIR156f* in rice shoot was specifically expressed by *D18* promoter and a semi-dwarf transformant with more effective panicles and high grain yield was identified (Fig. 2a, Table 2).

Ratooning can achieve ‘one crop with two harvests’ in rice cultivation and is an important measure to increase rice grain yield and to reduce production costs, especially under the current situation of rising costs of labor force. After the harvest of seasonal rice, fast and simultaneous outgrowth of axillary buds is crucial because late ratooning will usually encounter the low temperature stress. In this study, an important finding is the improvement of ratooning performance by manipulating the expression of *osa-MIR156f*. It was found that the axillary buds in basal node would outgrow in advance before the harvest of seasonal rice in D18–11, while the axillary buds of the wild type would largely remain dormant at the same time (Fig. 6b). New tillers would quickly outgrow from the basal and the last one to two nodes in D18–11, thus the ratooning rice grain yield was significantly higher than that of the wild type. Therefore, specific expression of *osa-MIR156f* in the shoot could be applicable in future genetic breeding for shaping plant architecture and yield traits both in seasonal and ratooning rice.

## Conclusion

In this study, *osa-MIR156f* was specifically expressed in the rice stem under the control of the promoter *D18*. The transgenic line showed moderate height and more effective tillers. Moreover, the grain yield was significantly increased both in seasonal and ratooning rice. The controlling expression of *osa-MIR156f* may have the great potential of application in rice genetic breeding. In addition, we found that osa-miR156f down-regulated the expression of tillering regulators, such as *TB1* and *LAX1*. The expression of *D10*, *D27* and *D53,* being involved in the biosynthesis and signaling of strigolactone (SL), decreased in the stem of the D18–11 transgenic line.

## Methods

### Plant Materials

The cultivated rice *O. sativa* spp. *japonica* cv. Nipponbare was used for *osa-MIR156f* transformation. Rice plants of the transgenic lines and the wild type were grown in pots in a net-house under natural conditions, and necessary management practices were provided as required for proper growth. At the full heading stage, all stems of each hills were removed by hand to leave an approximately 10 cm of stubble, and sample pots were randomly selected to assess the ratooning performance.

### Plasmid Construction and Plant Transformation

The *osa-MIR156f* (Additional file 1: Supplemental file S1) was amplified through PCR from the Nipponbare genomic DNA by using the primers 5′-CGCCCACCTTTCTTCTCCCA-3′ and 5′-AAGGAGCAGTTAGATAATGGAG-3′. To generate *UBQpro::osa-MIR156f* in which the *osa-MIR156f* gene is driven by a *Ubiquitin1* promoter, a fragment of *osa-MIR156f* was cloned into the *Eco*R V site of pBluescriptSK+ (pBS) (designated as pBS-*MIR156f*) and subsequently sequenced. The maize *Ubiquitin1* promoter was collected from the plasmid pBS-pUbq by digesting with *Hin*d III/*Bam*H I. The pBS-*MIR156f* was digested by *Sal* I/*Bam*H I to obtain the fragment including *osa-MIR156f*. The pWM101 vector was digested by *Hin*d III/*Sal* I, and then ligated with *Ubiquitin1* promoter and *osa-MIR156f* to obtain *UBQpro::osa-MIR156f.* To generate *D18pro::osa-MIR156f* in which the *osa-MIR156f* gene is driven by the *D18* promoter (Additional file 1: Supplemental file S2), the maize *Ubiquitin1* promoter of *UBQpro::osa-MIR156f* was removed and replaced by a 2.1-kb segment of the *D18* promoter [46].

To generate the *osa-MIR156f pro::GUS* construct, 3 kb of the promoter region was predicted by using PlantCARE method (http://bioinformatics.psb.ugent.be/webtools/ plantcare/html/) and was cloned from Nipponbare genomic DNA. The promoter of *osa-MIR156f* (a 3 kb fragment in upstream region of *pre-miRNA156f*) was amplified by using the primers 5′-ATGGAATAAATGGCGCCGTGTAC-3′ and 5′-ACTGCCACCACCCAAAACCAAGA-3′, and then cloned into the *pKGWS7*-Gateway-GUS plasmid to generate the *osa-MIR156f pro::GUS* vector by an LR reaction. An *Agrobacterium tumefaciens* mediated transformation method as previously reported by Hiei and Komari [52] .

### Realtime-qPCR and Stem-Loop RT PCR

The leaves, basal nodes and roots respectively at the 3-week-old seedling stage and the tillering stage, the stem at elongation stage and the young spikes at the heading stage were used for gene expression analysis. Tissues from 5 individual plants were pooled. Total RNA was isolated from 100 mg of tissues using a Trizol reagent (Invitrogen) and treated with RNase-free DNase I (Invitrogen) according to the manufacturer’s instructions. Approximately 5 μg of high-quality RNA was used to synthesize first-strand cDNA using poly (dT) oligo primer according to the manufacturer’s instructions in M-MLV kit (Invitrogen). Eppendorf BioPhotometer Plus (Germany) was used to analyze RNA/cDNA quality and to quantify RNA/cDNA concentration. PCR was carried out in a reaction system with a total volume of 20 μL, which contained 0.2 μL cDNA, 0.2 μM each specific primer and SYBR Green I (Invitrogen) on a CFX96 system (BIO-RAD). The following programs were employed: pre-denaturing for 30 s at 95 °C, then amplification for 40 cycles including denaturation for 10 s at 95 °C, and annealing for 30 s at 60 °C. Both the tillering related genes and *pri-miR156f* were normalized to the internal rice *tubulin β-4* gene.

Stem-loop reverse transcription quantitative PCR (stem-loop RT PCR) [53] was employed to detect the mature osa-miR156f, and *U6* snRNA was used as an internal control. Reverse transcription in a 20 μL reaction system (containing 1 μg RNA, 1 μM each primer, 0.5 μL Invitrogen M-MLV reverse transcriptase) was conducted in an thermal cycler using a pulsed RT program as follows: incubate for 30 min at 16 °C, 60 cycles at 30 °C for 30 s, 42 °C for 30 s and 50 °C for 1 s, followed by incubation at 85 °C for 5 min to inactivate the reverse transcriptase. cDNA concentration was quantified by using Eppendorf BioPhotometer Plus (Germany) and same amount of cDNA was used as the template for the next Stem-loop PCR. Stem-loop PCR was carried out in a reaction system with a total volume of 20 μL, which contained SYBR Green I (Invitrogen) on a CFX96 system (BIO-RAD). The following programs were employed: pre-denaturing for 30 s at 95 °C, then amplification for 40 cycles including denaturation for 10 s at 95 °C, and annealing for 30 s at 60 °C.

For melting curve analysis, reaction mix was denatured samples at 95 °C, then cooled to 60 °C. The amplification specificity was monitored by collecting fluorescence signals continuously from 60 °C to 95 °C at 0.5 °C per second. The ΔΔCt method was used to calculate the gene relative expression. The primers used were listed in the Additional file 1: Supplemental file S3. Three independent biological replicates were analyzed (*n* = 3).

### GUS Staining

Five *osa-MIR156fpro::GUS* transgenic plants were used for GUS staining analysis. The tissues for analysis including leaves and roots respectively collected at the seedling, the tillering and the heading stage. The sampling dates for additional tissues were as follows: shoot apexes at the 3-week-old seedling stage, axillary buds at the tillering and heading stage, basal nodes at the tillering and heading stage, stem segments at the stem elongation and flowering stage, spikes at the heading stage, and stamen at the flowering stage. These tissues were washed three times with 100 mM phosphate buffer (pH 7.0), and then incubated in a staining solution [100 mM phosphate buffer (pH 7.0), 10 mM EDTA, 2 mM 5-bromo-4-chloro-3-indolyl-b-GlcA, 5 mM K_4_Fe(CN)_6_, 5 mM K_3_Fe(CN)_6_, and 0.2% Triton X-100] for 24 h at 37 °C. The stained tissues were finally observed after soaked in 90% alcohol for 24–48 h [54].

### Strigolactone Extraction and Determination

Rice tissue was ground in liquid nitrogen and about 200 mg well ground sample was loaded to a 2.0 mL vial. After the addition of 1.0 mL of acetone (HPLC grade, Tedia), the homogenates were mixed by stirring in an ultrasonic bath and stored overnight at − 20 °C. After being centrifuged at 15,200 g for 10 min, the supernatant was collected and then vacuumed to dryness by RCT-60 concentrator (Jouan). Dried extract was dissolved in 300 μL of methanol (HPLC grade, Merck) solution and then passed through a Sep-Pak C_18_ cartridge (Waters, USA). The cartridge was eluted with 1500 μL of 80% methanol, and the eluate was once again vacuum-dried. After re-dissolving in 50 μL of 80% methanol, 10 μL of sample solution was injected into the liquid chromatography-tandem mass spectrometry system (LC-MS/MS 8030 plus, Shimadzu, Japan).

Liquid chromatography was performed using a 2.0 i.d. mm × 75 mm Shim-pack XR-ODSI column (2.2 μm, Shimadzu) at a column temperature of 40 °C.The mobile phase comprising solvent A (0.1% v/v aqueous formic acid) and solvent B (100% v/v acetonitrile) was employed in a gradient mode simply described as “min/%/%,” i.e., retention time/concentration of A/concentration of B. The gradient program for SL was 0/90/10, 12/0/100, 13/0/100, 14/90/10. The flow rate of the mobile phase was 0.25 mL·min^− 1^.

The mass system was set to multiple-reaction-monitoring mode using electrospray ionization in the positive ion mode. 5-dexoxystrigol (5-DS, Strigolab) was used as an external standard for SL analysis. Optimized mass operation conditions were employed as follows: nebulizing gas flow (1.5 L·min^− 1^), drying gas flow (12 L·min^− 1^), desolvation temperature (200 °C) and heat block temperature (500 °C). For 5-DS quantitive analysis, quadrupole 1 pre-bias of − 17 eV, quadrupole 3 pre-bias of − 22 eV, collision energy of − 17 eV, mass-to-charge ratio (m/z) of 331.1/216.2 were employed. The SL concentration was eventually calculated according to the calibration curve established by using 5-DS solution at different concentrations, and a good linear determination coefficients R^2^ (0.9967) of the calibration curve was obtained for all the analytes. Three independent biological replicates were analyzed.

### Morphological Observations and Yield Related Traits Investigation

Plant height, leaf length and leaf width were measured weekly from the day after transplanting, and tiller number was investigated at a 15-day interval from 30 days after transplanting. The yield related traits, including tiller number per plant, main panicle length (cm), spikelet number per panicle, filled grain number per panicle, 1000-grain weight (g), and grain yield per plant (g) were recorded after harvest. For the ratooning performance, plant height, number of tillers and total grain weight per hill were measured. Three independent biological replicates were analyzed.

### Root Number and Root Activity Analysis

After the seasonal rice harvest, old roots were cut off from the rice stubble and the rice stubble was replanted in soil. After culturing for 7 days, the number of the regenerated roots was counted. A TTC (2,3,5-triphenyte-trazolium chloride) method [55] was employed to analyze the root activity of regenerated roots. TTC (Sigma) was dissolved in 100 mM sodium phosphate buffer (pH 7.0) to a final concentration of 0.6% (w/v). Root segments about 1 cm to 3 cm in length, were collected and washed with sterile water for 10 min. The root segments were transferred into 1 mL of TTC solution and incubated for 1 h at 37 °C in the dark. The TTC solution was removed and the root segments were rinsed once with deionized water. For the extraction process, the root segments were incubated overnight at room temperature in 2 mL of 95% (v/v) ethanol. The reduction of TTC was expressed as the absorbance of the extracted solutions at 520 nm in a spectrophotometer. Three independent biological replicates were analyzed.

## Supplementary information

**Additional file 1.** Supplemental file S1-S6.

## Data Availability

The datasets measured and analyzed during the study are available from the corresponding authors upon reasonable request.
