# Supplementary material for: Manipulating osa-MIR156f Expression by D18 Promoter to Regulate Plant Architecture and Yield Traits both in Seasonal and Ratooning Rice
Source: Biol Proced Online. 2019 Nov 4;21:21. doi: 10.1186/s12575-019-0110-4 (PMC6827258; doi:10.1186/s12575-019-0110-4)

Supplemental file S1: Sequence of *Osa-MIR156f* and Osa-miR156f

Osa-MIR156f :

ccgtcgccgtcatcctctcgccgatcctcctctccgcctcgactccggcgacctcctcgccggtgagccccagcgccctcaagaacaa
cctcagcaactcgtcggctagccgccgcatctccttgtgaaactcctccatcacgtcactgcgtatattatacgtagatcaatacgtagata
tatacgtagaggtagatatatacgtagagtgtatgtgcatggaatgcatgggagagtatatatgtatatataccagaagaggaggtagtc
gtcgccggacttgggccagaggcggcggagctcggagcggagggaggaaggggagaaggtgtagccctcggaccacatgagct
tggagaagaaggaggagatgggcggcgagccgtagccgcagggctcgccggggccgcggacggcgcgcatcttctccgacgcc
ggcagggagaacacgcgggcgacgcgctcctcgacgcgcgacagcagcgccgccggcacgccgtgcccgaccagaaggaacg
cgccccactgctccgccgcccgcgccacccgcgccgccgcgtcgcccgccccgacgtccaccaccggcaccgcgtcctcgccgc
cgccgccgccgccgtccaccaccgggtggtcgtccagccccggccacgcgtgcgtctccggcacccgcctcgccgcccggaagtc
gaagcagagcgggttcttcaagtgcgacggcgtcggcatcgcgacgacgacgacgatgaccagagagcaacacactcgcgcgctc
gctcgtctcgtctggcagaaagccagtaacaataacaacactaatttttctctctatataaatttttctatgactttacaatagtggaatgagt
agtaaagtgagacgagtgtaagaactggacaagtacaagggagaggtatatatagggagagaagttttggagcgtagtggaagaaaa
aaaaagagagaattaattggtgaataaatcagtgatgaagaattgaaatggatcattaattaatagatttggagaggaaaaaatgtggtga
ttgggacagagggtttggagtgatgtggagcacacaaggccaccagcttagctacacaacacaaatctagctagtatgctcatcaccac
cccctggctggctagtggcactgccacccaattaataacacacatattcatacaaattaagttatgttatagtaaagtaaaatcacaatgtg
aaataagcaccagagtaattttaattatcacattacataaaacagattaggtaattaaacataggaatttcacaggaacagtagcgtcctc
cgttgccgtggtgttttttttaattcgtctgtctgctttagaaccaaacattctcacatgcagccacttctaattaattatgattaccgtataattc
tcatacacgtcgaatttcattcgatattttactttcagtagcaagatcttcatgctagttcccatatacagtactacatccgtttcaggttataag
acgtcttgactttagtcaaaatcaaactgctttaagtttgactaattttatagaaaaagtagtaatatttttaactcaagacaaatttattatgaaa
atatatttaattaactaatttagtattataaatattattatatttgtctataaaattagtcaaacttgaaacaacatgactttgactaaagtcaaatc
attttataatctgaaacgataggattacgtcctaatttgtcgagcacagtacaattaccaagattcaaaccgtgttatctacttcttctttttcaa
tatatttactaagaagactaaccttatattacaactacaaaaataaacaaaacaaaacaaatataaacagtaaattgtttagaaaacagcac
acagattctagaacagcaaacaagagaaaagacagcactagtctgcagccacgagtaaaaacagaggacagaggccatgatcatca
tcatccatccatccatgaatggttgctaatacattacaaaattaacacagagacagagagagagagagagagagagagagagagaga
ggatgcattgggtctggaggtaggcggcg

Osa-miR156f:

cgcccacctttcttctcccactccggcggcacatctccgccgccggccggtcagccggcagctagtggctctctcgctcctatgtggct
atgtctgtctacctaatggttttctgttttcttggttttgggtggtggcagttgacagaagagagtgagcacacagcggccagactgcatcg
atctatcaatcttcccttcgacaggatagctagatagaaagaaagagaggccgtcggcggccatggaagagagagagagagagaga
tgaaatgatgatgatgatacagctgccgctgcgtgctcacttctctttctgtcagctctccctgcagcatgcacgctgccacagccacact
ctctccattatctaactgctcctt

Supplemental file S2: Sequence of *D18* promoter:

ccgtcgccgtcatcctctcgccgatcctcctctccgcctcgactccggcgacctcctcgccggtgagccccagcgccctcaagaacaa
cctcagcaactcgtcggctagccgccgcatctccttgtgaaactcctccatcacgtcactgcgtatattatacgtagatcaatacgtagata
tatacgtagaggtagatatatacgtagagtgtatgtgcatggaatgcatgggagagtatatatgtatatataccagaagaggaggtagtc
gtcgccggacttgggccagaggcggcggagctcggagcggagggaggaaggggagaaggtgtagccctcggaccacatgagct
tggagaagaaggaggagatgggcggcgagccgtagccgcagggctcgccggggccgcggacggcgcgcatcttctccgacgcc
ggcagggagaacacgcgggcgacgcgctcctcgacgcgcgacagcagcgccgccggcacgccgtgcccgaccagaaggaacg
cgccccactgctccgccgcccgcgccacccgcgccgccgcgtcgcccgccccgacgtccaccaccggcaccgcgtcctcgccgc
cgccgccgccgccgtccaccaccgggtggtcgtccagccccggccacgcgtgcgtctccggcacccgcctcgccgcccggaagtc
gaagcagagcgggttcttcaagtgcgacggcgtcggcatcgcgacgacgacgacgatgaccagagagcaacacactcgcgcgctc
gctcgtctcgtctggcagaaagccagtaacaataacaacactaattttttctctctatataaatttttctatgactttacaatagtggaatgagt
agtaaagtgagacgagtgtaagaactggacaagtacaagggagaggtatatatagggagagaagttttggagcgtagtggaagaaaa
aaaaagagagaattaattggtgaataaatcagtgatgaagaattgaaatggatcattaattaatagatttggagaggaaaaaatgtggtga
ttgggacagagggtttggagtgatgtggagcacacaaggccaccagcttagctacacaacacaaatctagctagtatgctcatcaccac
cccctggctggctagtggcactgccacccaattaataacacacatattcatacaaattaagttatgttatagtaaagtaaaatcacaatgtg
aaataagcaccagagtaatttttaattatcacattacataaaacagattaggtaattaaacataggaatttcacaggaacagtagcgtcctc
cgttgccgtggtgtttttttttaattcgtctgtctgctttagaaccaaacattctcacatgcagccacttctaattaattatgattaccgtataattc
tcatacacgtcgaatttcattcgatattttactttcagtagcaagatcttcatgctagttcccatatacagtactacatccgtttcaggttataag
acgtcttgactttagtcaaaatcaaactgctttaagtttgactaattttatagaaaaagtagtaatatttttaactcaagacaaatttattatgaaa
atatatttaattaactaatttagtattataaatattattatatttgtctataaaattagtcaaacttgaaacaacatgactttgactaaagtcaaatc
attttataatctgaaacgataggattacgtcctaatttgtcgagcacagtacaattaccaagattcaaaccgtgttatctacttcttctttttcaa
tatatttactaagaagactaaccttatattacaactacaaaaataaacaaaacaaaacaaatataaacagtaaattgtttagaaaacagcac
acagattctagaacagcaaacaagagaaaagacagcactagtctgcagccacgagtaaaaacagaggacagaggccatgatcatca
tcatccatccatccatgaatggttgctaatacattacaaaattaacacagagacagagagagagagagagagagagagagagagaga
ggatgcattgggtctggaggtaggcggcg

Supplemental file S3: Primer used in paper and specificity analysis

primer	primer sequences	gene	extended name	accession number	reference
501TB1-F	GCCGGATGCAAGAAATCTCG	*OsTB1*	TEOSINTE BRANCHED 1	XM_015774751(LOC4333856)	Takeda *et al.* 2003
502TB1-R	TCAGCAGTAGTGCCGCGAA				
503D14-F	CGCCTTCGTCGGCCACTCCGT	*OsDWARF14*	DWARF 14	XM_015775914(LOC4331983)	
504D14-R	TCGAACCCGCCGTGGTAGTC				
505D27-F	GCGTCGCAAAGCAGAATCAT	*OsDWARF27*	DWARF 27	XM_015759767(LOC107276001)	
506D27-R	GCTGGTGTTGTGGGCAAAAA				
509MADS57-F	ACAAGCTTCAGGCCTGTGAA	*OsMADS57*	MADS-box transcription factor 57-like	XM_015771309(LOC4330621)	
510MADS57-R	TCTGGAGCAGCTGTTTTGC				
511LAX1-F	TGCTACTCGGTGTGCAATGT	*OsLAX1*	LAX PANICLE 1-like	XM_015763095(LOC4327431)	
512LAX1-R	GGACCATGACCAACCGGTAA				
513RFL1-F	TGGGAGTTTCCTGTGGTGTTG	*OsRFL1*	RICE FLORICAULA/LEAFY 1	XM_015779869(LOC4336857)	Wang *et al.* 2015
514RFL1-R	CCATCCAGATCGCAAACATG				
515RCN1-F	GCCATCCTTCAGGGACCATT	*OsRCN1*	Rice TFL1/CEN homolog1	XM_026021171(LOC4349798)	
516RCN1-R	CTGCATTTCTCAGCGCCTCC				
521D53-F	AATCTGGGTGCTGAGTGCAT	*OsD53*	DWARF 53	XM_015764885(LOC4351252)	
522D53-R	GGGCCCTTGTCCTCTGATTT				
523D10-F	CGTGGCGATATCGATGGT	*OsD10*	DWARF 10	XM_015787274(LOC4326177)	Zhou *et al.* 2013
524D10-R	CGACCTCCTCGAACGTCTT				

525SPL3-F	AAAGTCATCGTTGCGGGTCT	*OsSPL3*	Squamosa Promoter-binding-Like protein 3	XM_015771400(LOC9270639)	
526SPL3-R	GTTGCCTTGCATTTAAGGAAATTG				
527SPL7-F	GTTTGACGACGCCAAGAAG	*OsSPL7*	Squamosa Promoter-binding-Like protein 7	XM_015779858(LOC4336597)	Wang *et al.* 2015
528SPL7-R	GGCTCTAGCCGATGACAGA				
529SPL13-F	GTGCTATGCTGGAGAGGCAT	*OsSPL13*	Squamosa Promoter-binding-Like protein 13	XM_015789929(LOC9270355)	
530SPL13-R	GTTTCTGTTGGGCAAAGGCA				
531SPL14-F	TAGCCATCATGCCCACTTC	*OsSPL14*	Squamosa Promoter-binding-Like protein 14	XM_026027598(LOC4345998)	Wang *et al.* 2015
532SPL14-R	AGACCAATCCATCGTGTTG				
403-tubF	TACCGTGCCCTTACTGTTCC	*Tubulin beta-4*		XM_015794238(LOC4327550)	Kim *et al.* 2003
404-tubR	CGGTGGAATGTCACAGACAC				
stem-MiR156	GTCGTATCCAGTGCAGGGTCCGAG GTATTCGCACTGGATACGACgtgctc				Varkonyi-Gasic *et al.* 2007
miR156-forward	gcggcggTGACAGAAGAGAGT				
Universal Reverse Primer	GTGCAGGGTCCGAGGT			NC_029263	
94ospri156F	CTTCCCTTCGACAGGATAGC				
95ospri156R	AGCGGCAGCTGTATCATCA				
OsU6-F	TACAGATAAGATTAGCATGGCCCC	U6 snRNA			Wang *et al.* 2015
OsU6-R	GGACCATTTCTCGATTTGTACGTG				

Reference in Supplemental file S3

1. Takeda T, Suwa Y, Suzuki M, Kitano H, Ueguchi-Tanaka M, Ashikari M, Matsuoka M, Ueguchi C. The *OsTB1* gene negatively regulates lateral branching in rice. *Plant J.* 2003, 33(3):513-520.

2. Wang, L., Sun, S., Jin, J., Fu, D., Yang, X., Weng, X., Xu, C., Li, X., Xiao, J., Zhang, Q. Coordinated regulation of vegetative and reproductive branching in rice. *Proc. Nat. Acad. Sci.* 2015, 112(50) : 15504-15509.

3. Zhou F, Lin Q, Zhu L, Ren Y, Zhou K, Shabek N, Wu F, Mao H, Dong W, Gan L, Ma W, Gao H, Chen J, Yang C, Wang D, Tan J, Zhang X, Guo X, Wang J, Jiang L, Liu X, Chen W, Chu J, Yan C, Ueno K, Ito S, Asami T, Cheng Z, Wang J, Lei C, Zhai H, Wu C, Wang H, Zheng N, Wan J. D14-SCF^{D3}-dependent degradation of D53 regulates strigolactone signaling. Nature. 2013, 504(7480):406-10

4. Varkonyi-Gasic, E., Wu, R., Wood, M., Walton, E.F., Hellens, R.P. Protocol: a highly sensitive RT- PCR method for detection and quantification of microRNAs. Plant Methods. 2007, 3(1) : 12.

5. Kim BR, Nam HY, Kim SU, Kim SI, Chang YJ. Normalization of reverse transcription quantitative-PCR with housekeeping genes in rice. *Biotechnol Lett*. 2003, 25(21):1869-72.

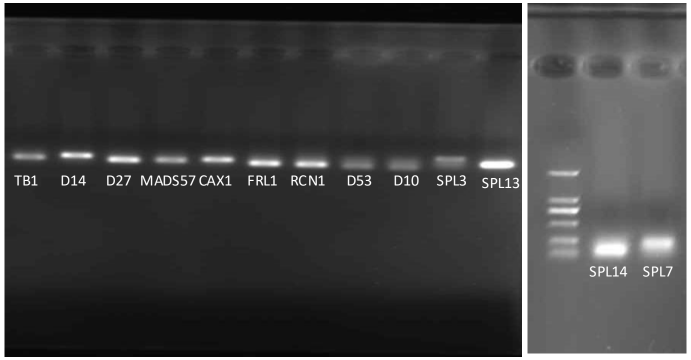

Specificity analysis of RT-qPCR and Stem-loop PCR production

Supplemental file S4: GUS analysis of rice tissues

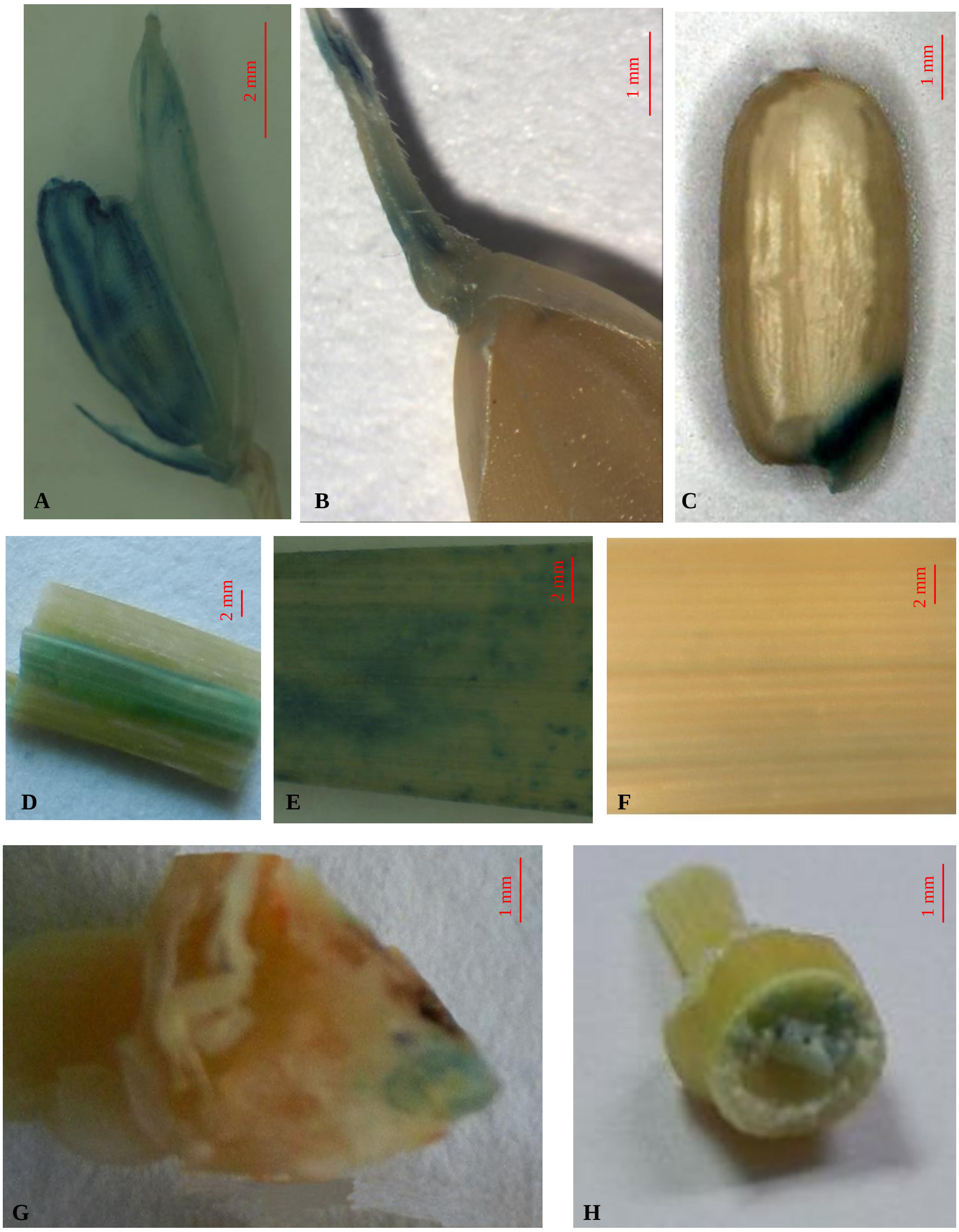

A) grain at milking stage; B) mature grain; C) germinating grain; D) leaf lamina at tillering stage; E) flag leaf segment at heading stage; F) leaf segment at mature stage; G) basal node and roots at tillering stage; H) stem node at stem elongation stage.

Supplemental file S5: Phenotypes of *D18pro::osa-MIR156f* independent transgenic lines. A: 30 day-old seedlings; B: 50 day-old plants; C: Panicle phenotype of transgenic lines and WT in seasonal rice; D: Ratooning rice of D18-3 and WT

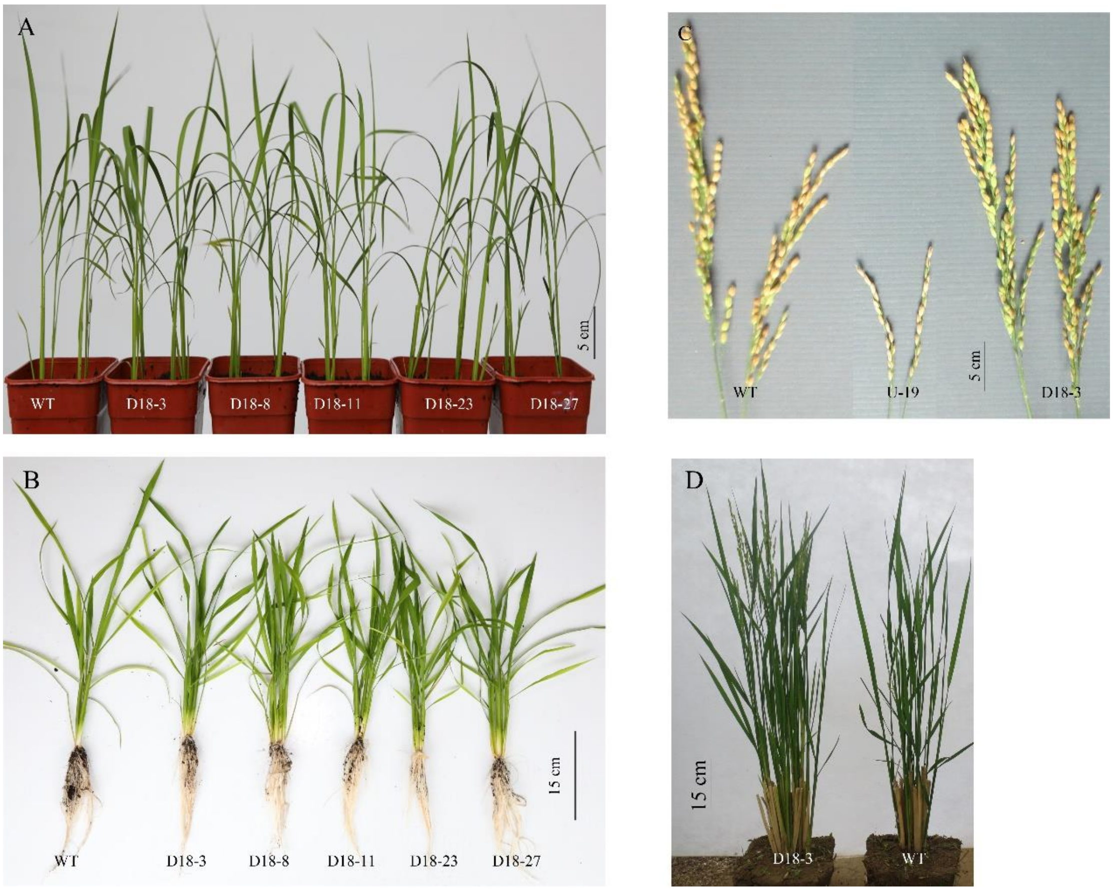

Supplemental file S6: Expression analysis of tillering and branch related genes

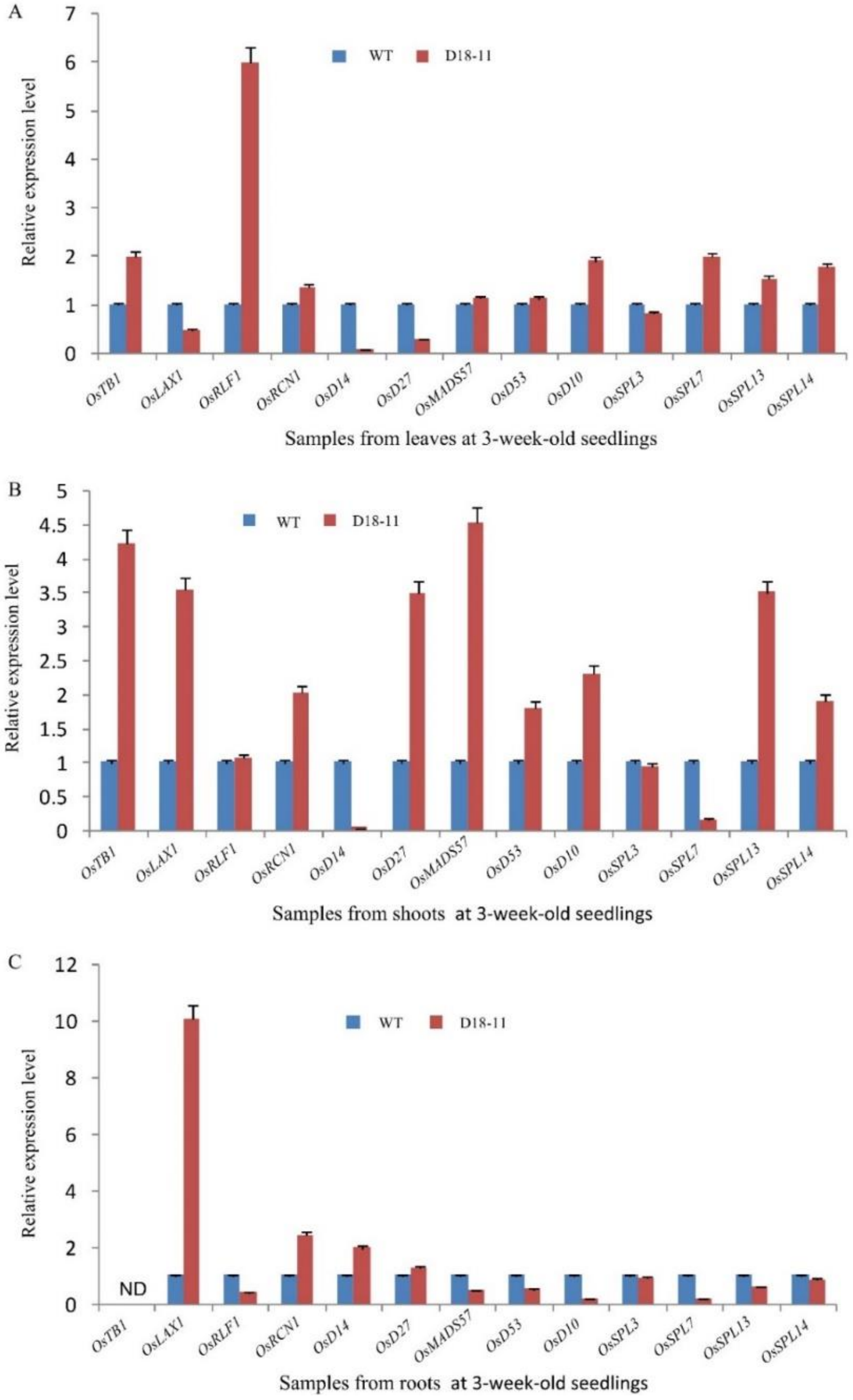

Supplement: Supplementary file 1 — Additional file 1. Supplemental file S1-S6. [file 12575_2019_110_MOESM1_ESM.pdf]
